# Supplementary material for: Investigating the Contribution of Molecular‐Enriched Functional Connectivity to Brain‐Age Analysis
Source: Hum Brain Mapp. 2026 Sep 1;47(13):e70627. doi: 10.1002/hbm.70627 (PMC13535138; doi:10.1002/hbm.70627)
Supplement: Supplementary file 1 [file HBM-47-e70627-s001.zip › supplementary/supplementary.pdf]

Supplementary Material

Subjects excluded

Quality control of raw images

Automated MRIQC screening flagged a subset of scans with poor signal-to-noise ratio, motion artifacts, or other anomalies; those participants were removed before preprocessing. In total, 61 individuals were excluded: 51 because of structural issues and 10 because of functional issues. The dataset-specific counts are listed in Supplementary Table S1.

Preprocessing outputs

During structural and functional MRI preprocessing, a small additional fraction of scans failed because of unrecoverable software errors or excessive motion (Supplementary Table S2). Specifically, *fMRIPrep* terminated early for five HCP-Aging participants, whereas no such failures occurred in Cam-CAN or NKI-RS. Subsequent denoising with XCP-D led to the exclusion of participants whose residual time series contained more than one-third of volumes with framewise displacement exceeding 0.5 mm. Finally, one subject was excluded from the Cam-CAN sample because `react-fmri` did not run successfully on it. Table S2 details these counts: in total, 165 participants were removed at the preprocessing stage, leaving 2128 individuals for downstream analyses. This number decreases to 2120 after the exclusion of HCP-Aging participants over 90 years old: this final sample constitutes the dataset used for this study.

Molecular-enriched functional connectivity

Only one run failed (subject from Cam-CAN) and thus that participant was excluded from the analysis.

Table S1: Participants removed after MRIQC

**Dataset**	**Structural (T1w)**	**Functional (rs-fMRI)**	**Total**
Cam-CAN	3	2	5
HCP-Aging	25	8	33
NKI-RS	23	0	23
**Total**	**51**	**10**	**61**

Table S2: Participants excluded during preprocessing.

**Dataset**	**fMRIPrep**	**XCP-D**	**REACT**	**Total**
Cam-CAN	0	20	1	21
HCP-Aging	5	79	0	84
NKI-RS	0	60	0	60
Total	5	159	1	165

Dataset differences in motion-related quality metrics

To assess potential differences in data quality across datasets, we quantified motion-related image quality metrics derived from MRIQC, including mean framewise displacement (FD) and standardized DVARS, in the final quality-controlled sample.

Group-level differences across datasets were evaluated using Kruskal–Wallis tests, due to the non-Gaussian distribution of motion metrics. Significant differences were observed for both FD and DVARS (Supplementary Table S3). Post-hoc pairwise comparisons were performed using Dunn's test with false discovery rate (FDR) correction (Supplementary Table S5). Descriptive statistics for each dataset are reported in Supplementary Table S4.

Overall, HCP-Aging exhibited lower motion levels compared to Cam-CAN and NKI-RS, while Cam-CAN and NKI-RS showed partially overlapping distributions (Supplementary Figure S1). These between-dataset differences in rs-fMRI quality provide one plausible source of the cross-cohort variability observed in single-transporter prediction performance, although this analysis was exploratory and not designed to isolate a causal contribution of QC alone.

All datasets were processed using the same preprocessing pipeline and parameters. Additionally, subjects with excessive motion were excluded, defined as having more than one-third of volumes exceeding a framewise displacement threshold of 0.5 mm during denoising. These steps ensured a consistent and conservative quality-controlled sample across datasets.

Table S3: Kruskal–Wallis tests assessing between-dataset differences in motion-related quality metrics in the final quality-controlled sample.

Metric	H statistic	p-value
Mean FD	395.47	1.33×10^{-86}
Standardized DVARS	1038.55	3.04×10^{-226}

Table S4: Descriptive statistics of motion-related quality metrics across datasets in the final quality-controlled sample. Values are reported as mean ± SD and median [Q1, Q3].

Metric	Dataset	N	Mean ± SD	Median [Q1, Q3]
Mean FD	Cam-CAN	626	0.310 ± 0.145	0.276 [0.202, 0.385]
	HCP-Aging	607	0.180 ± 0.058	0.173 [0.138, 0.216]
	NKI-RS	894	0.260 ± 0.104	0.237 [0.184, 0.318]
Standardized DVARS	Cam-CAN	626	1.180 ± 0.093	1.184 [1.126, 1.234]
	HCP-Aging	607	1.025 ± 0.039	1.027 [1.003, 1.049]
	NKI-RS	894	1.148 ± 0.070	1.151 [1.103, 1.192]

Table S5: Pairwise between-dataset comparisons for motion-related quality metrics using Dunn's test with FDR correction.

Metric	Comparison	FDR-corrected p-value
Mean FD	Cam-CAN vs HCP-Aging	1.46×10^{-80}
	Cam-CAN vs NKI-RS	3.94×10^{-8}
	HCP-Aging vs NKI-RS	4.78×10^{-52}
Standardized DVARS	Cam-CAN vs HCP-Aging	3.15×10^{-195}
	Cam-CAN vs NKI-RS	6.29×10^{-9}
	HCP-Aging vs NKI-RS	2.37×10^{-155}

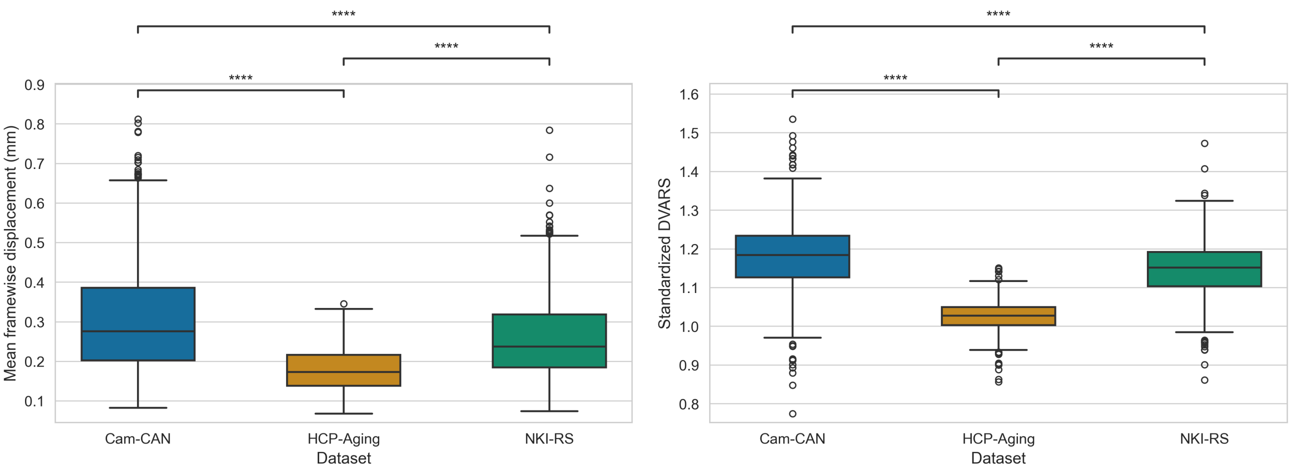

Figure S1: Distribution of motion-related quality metrics across datasets in the final quality-controlled sample. Left: mean framewise displacement (FD). Right: standardized DVARS. Boxes represent median and interquartile range; whiskers extend to 1.5× IQR. Statistical annotations indicate significant pairwise differences based on Dunn's post-hoc tests with FDR correction. These exploratory analyses indicate that the three cohorts differed in their rs-fMRI quality profiles.

Residual-level comparison of structure-only vs. multimodal models (common-parcellation analysis)

To evaluate whether the multimodal model (multimodal features (M_{Multi})) improves prediction beyond the structure-only model (structural morphometric features (M_{Struct})) in the DKT common-parcellation analysis, we compared participant-level out-of-sample residuals across the 10 repetitions of the outer 10-fold cross-validation using matched splits. For each repetition and participant, we computed the paired difference in absolute error, $\Delta|e| = |\widehat{\text{age}}_{\text{M}_{\text{Struct}}} - \text{age}| - |\widehat{\text{age}}_{\text{M}_{\text{Multi}}} - \text{age}|$, so that positive values indicate lower error under M_{Multi}. Supplementary Table S6 reports repetition-wise mean paired improvements, bootstrap confidence intervals across participants, paired standardized effect sizes (Cohen's d_z), and paired sign-flip permutation tests.

Table S6: Residual-level paired comparison between M_{Struct} and M_{Multi} in the common-parcellation (DKT) analysis.

Repetition	$\Delta|e|$ [years]	95% CI	Cohen's d_z	% improved	p (one-sided)	p (two-sided)
1	0.169	[0.041, 0.297]	0.057	52.4	0.0043	0.0097
2	0.196	[0.072, 0.319]	0.068	53.7	0.0010	0.0014
3	0.195	[0.072, 0.308]	0.068	52.7	7.00×10^{-4}	0.0015
4	0.225	[0.100, 0.346]	0.077	54.3	3.50×10^{-4}	4.00×10^{-4}
5	0.232	[0.105, 0.354]	0.079	53.4	1.50×10^{-4}	2.00×10^{-4}
6	0.202	[0.079, 0.328]	0.069	53.9	8.00×10^{-4}	0.0017
7	0.221	[0.097, 0.352]	0.076	53.4	4.00×10^{-4}	4.00×10^{-4}
8	0.244	[0.117, 0.382]	0.082	54.8	$< 1/(N_{\text{perm}} + 1)$	2.00×10^{-4}
9	0.185	[0.059, 0.310]	0.063	54.4	0.0017	0.0039
10	0.207	[0.082, 0.333]	0.070	54.0	5.50×10^{-4}	8.50×10^{-4}
**Across reps**	0.208 ± 0.023	0.191–0.224	0.071	53.7 ± 0.7	0.0043	0.0097

$\Delta|e|$ denotes the participant-level paired difference in out-of-sample absolute error, computed as $|\widehat{\text{age}}_{\text{M}_{\text{Struct}}} - \text{age}| - |\widehat{\text{age}}_{M_{Multi}} - \text{age}|$; positive values indicate lower error for M_{Multi}.
Within each repetition of the outer 10-fold cross-validation, permutation p-values were obtained with a paired sign-flip test on $\Delta|\Delta\text{age}|$ (one-sided: mean > 0). A reported value of $< 1/(N_{\text{perm}} + 1)$ indicates that no permuted replicate exceeded the observed statistic.
95% CIs for repetitions are bootstrap CIs across participants.
"Across reps" p-values summarize robustness conservatively as the maximum permutation p-value across repetitions (one-sided and two-sided, respectively).

Sensitivity to harmonization within the common-parcellation setting

To directly assess whether harmonization materially affects the Desikan–Killiany-Tourville atlas (DKT)-based comparison, we repeated the merged-cohort DKT analysis without combating batch effects (ComBat) harmonization using the same modeling pipeline (Table S7). Performance was highly similar with and without ComBat. For M_{Struct}, MAE was 6.26 without harmonization and 6.02 years with ComBat. For M_{Multi}, MAE was 6.16 without harmonization and 5.81 years with ComBat, with a corresponding increase in R^2 from 0.819 to 0.830. Effects on single-transporter models were mixed (e.g., norepinephrine transporter (NET) decreased after harmonization), but the overall ranking of feature sets and the multimodal advantage were preserved. Overall, these results indicate that the common-parcellation conclusions are robust to whether harmonization is applied, while ComBat provides modest additional benefit for the structural and multimodal models.

Feature	Harmonization	R	R^2	MAE	RMSE	ρ
DAT	No harm	0.545	0.292	12.476	15.184	-0.814
	ComBat	0.532	0.267	12.064	15.272	-0.774
NET	No harm	0.580	0.330	12.005	14.768	-0.776
	ComBat	0.477	0.214	12.610	15.814	-0.828
SERT	No harm	0.571	0.323	12.164	14.848	-0.798
	ComBat	0.517	0.260	12.281	15.344	-0.811
M_{Struct}	No harm	0.902	0.812	6.257	7.815	-0.414
	ComBat	0.908	0.824	6.017	7.489	-0.400
M_{Mol}	No harm	0.670	0.443	10.887	13.468	-0.705
	ComBat	0.657	0.427	10.453	13.494	-0.707
M_{Multi}	No harm	0.906	0.819	6.161	7.684	-0.415
	ComBat	0.912	0.830	5.809	7.343	-0.398

Table S7: **Sensitivity analysis of ComBat harmonization in the DKT common-parcellation setting.** Metrics reported: r (Pearson correlation), R^2 (coefficient of determination), mean absolute error (MAE), root-mean-squared error (RMSE), and ρ (age-bias correlation, computed as the correlation between brain-age delta and chronological age). Feature blocks correspond to molecular-enriched features (M_{Mol}), M_{Struct}, and M_{Multi}. Each value is the mean (averaged over the 10 folds and 10 repetitions) from a 10×10-fold cross-validation (CV) (100 trained models per configuration).
